# Opacity of Cornea Produced by Cocaine

**Published:** 1886-03

**Authors:** 


### Opacity of Cornea Produced by Cocaine.

Fletcher Wilson reports two cases of corneal opacity due to the instillation of cocaine. Fano claims that this accident will not happen unless the action of the cocaine is kept up for a long time, at least more than twenty-four hours.

